# The effects of clioquinol in morphogenesis, cell membrane and ion homeostasis in *Candida albicans*

**DOI:** 10.1186/s12866-020-01850-3

**Published:** 2020-06-16

**Authors:** Zimeng You, Chaoliang Zhang, Yuping Ran

**Affiliations:** 1 Department of Dermatovenereology, West China Hospital, Sichuan University, No. 37, Guo Xue Xiang, Wuhou District, Chengdu, 610041 Sichuan Province China; 2 State Key Laboratory of Oral Diseases, West China Stomatology Hospital, Sichuan University, Chengdu, 610041 Sichuan China

**Keywords:** Clioquinol, *Candida albicans*, Yeast-hyphae transition, Biofilm formation, Cell membrane, Ion homeostasis

## Abstract

**Background:**

*Candida albicans* is the most prevalent opportunistic fungal pathogen. Development of antifungals with novel targets is necessary for limitations of current antifungal agents and the emergence of drug resistance. The antifungal activity of clioquinol was widely accepted while the precise mechanism was poorly understood. Hence, we aimed to seek for the possible mechanism of clioquinol against *Candida albicans* in the present study.

**Results:**

Clioquinol could inhibit hyphae formation in a concentration-dependent manner in multiple liquid and solid media. The concentration and time-dependent anti-biofilm activities were observed in different incubation periods quantitatively and qualitatively. Further investigation found that clioquinol disrupted cell membrane directly in high concentration and induced depolarization of the membrane in low concentration. As for the influence on ion homeostasis, the antifungal effects of clioquinol could be reversed by exogenous addition of metal ions. Meanwhile, the minimum inhibitory concentration of clioquinol was increased in media supplemented with exogenous metal ions and decreased in media supplemented with exogenous metal chelators. We also found that the cellular labile ferrous iron level decreased when fungal cells were treated with clioquinol.

**Conclusion:**

These results indicated that clioquinol could inhibit yeast-hyphae transition and biofilm formation in *Candida albicans*. The effect on the cell membrane was different depending on different concentrations of clioquinol. Meanwhile, clioquinol could interfere with ion homeostasis as metal chelators for zinc, copper and iron, which was quite different with current common antifungal agents. All in all, clioquinol can be a new promising antifungal agent with novel target though more studies are needed to better understand the precise antifungal mechanism.

## Background

*Candida albicans* is a worldwide fungal pathogen in human beings, which can cause mucosal, cutaneous and life-threatening systemic infections, especially in immunosuppressed patients. By now, the available antifungal agents used in clinical work include polyenes, azoles, allylamines and echinocandins. The target of azoles, allylamines and polyenes was cell membrane while echinocandins inhibit fungal cell wall biosynthesis. However, the toxicity of the liver, kidney and high expense also limit the use of parts of these drugs, such as polyenes and echinocandins [1]. Hence, developing novel antifungal agents is necessary to treat the fungal infection effectively based on the above reasons and the increasing drug-resistance strains in the clinical work.

Clioquinol (5-chloro-7-iodoquinolin-8-ol, CQ) is an antimicrobial agent widely to treat multiple skin infections. In the 1950s–1970s, it was used as an oral anti-parasitic agent for the treatment and prevention of intestinal amebiasis. However, oral formulation of CQ was banned due to subacute myelo-opticneuorpathy (SMON) in Japanese patients in the 1970s. There are still controversies between the use of CQ and the occurrence of SMON. The reason for the neurological side effect is still not clear, but it is related to concomitant vitamin B deficiency and/or genetic susceptibility according to the previous studies [2, 3]. In fact, the topical formulations of CQ are still available for the treatment of topical fungal infections in clinical work.

Recently, CQ has reemerged for the treatment of non-infectious diseases including malignancies [4–6] and neurodegenerative diseases [2, 7]. The mechanism of CQ in treating this disease is related to its metal chelating property [4–7]. As for its antimicrobial activity, researchers confirmed the inhibition effect of CQ in the growth of *Candida spp.*, *Dermatophytes*, *Malassezia*, *Aspergillus* [8–10]. Some researchers also found that it could inhibit the growth of some bacteria [8, 11], which was not observed in our previous study [10].

Although the antifungal activity of CQ is widely accepted by researchers, the antifungal mechanism of CQ was not clear until now. Yan et al. [12] thought CQ induced G_2_/M cell cycle arrest through the up-regulation of TDH3 in *Saccharomyces cerevisiae*. While Pippi et al. [13, 14] found that CQ damaged the cell wall and inhibited the formation of pseudohyphae and biofilm in *Candida albicans*. However, the precise mechanism of CQ remains poorly understood.

A key virulence factor expressed by *C. albicans* that contributes significantly to its pathogenicity is the transition from yeast cells to hyphae or pseudohyphae and hyphae are critical for *C. albicans* to destroy host cells or tissues [15]. This cellular morphogenesis plays an important role in host tissues invasion, host immune escape and dissemination into circulation system [16]. Besides, the transition of *C. albicans* from yeast to hyphae is also related to biofilm formation [17, 18]. And *C. albicans* is the most common pathogen fungi that can form fungal biofilms, which are highly resistant to treatment with azole antifungals [19, 20]. Thus, inhibition of the yeast-to-hyphae transition and biofilm formation plays a key role in decreasing virulence of *C. albicans*.

Ion homeostasis was related to oxidative stress response, morphogenesis, drug resistance, cell wall integrity, and invasive growth in *C. albicans* and it could be novel targets of antifungal agents. Iron is the most abundant metal, which is involved in oxygen transport, tricarboxylic acid (TCA) cycle, DNA synthesis, etc. Zinc serves as a second messenger in various signaling pathways and a cofactor for various proteins [1]. Many Cu-containing enzymes have oxygen-related functions, such as superoxide dismutases [21]. And researchers found that several antifungal agents could inhibit fungal growth by influencing ion homeostasis.

In this study, we deeply investigated the effects of CQ in hyphae formation, biofilm formation and cell wall based on the results of Pippi et al. [13, 14]. Then we focused on the effects of CQ in the structure and function of the cell membrane. Based on the CQ’s affinity for metals [1], effect in ion homeostasis was also investigated to gain information about the possible mechanism.

## Results

### Clioquinol exhibited fungistatic and fungicidal activity for *C. albicans*

The minimum inhibitory concentration (MIC) of CQ, ketoconazole (KTZ), itraconazole (ITR), fluconazole (FLC), amphotericin B (AMB) and terbinafine (TBF) against *C. albicans* SC5314 was 1, 0.25, 0.5, 0.125, 1 and > 64 μg/ml, respectively. The results of other strains (*C. albicans* ATCC 10231 and 2 clinical strains) were reported before [10], which were similar to the results of *C. albicans* SC5314.

**Fig. 1:**
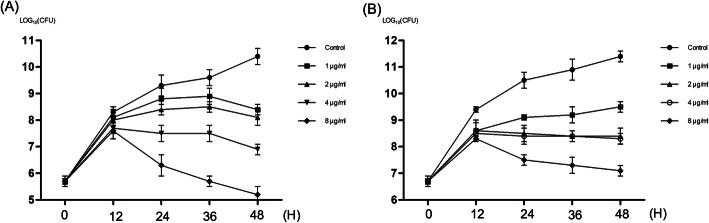
Time-kill curve of clioquinol against *Candida albicans*. The starting inoculum concentration of suspension was 5 × 10^5^ cells/ml (**a**) and 5 × 10^6^ cells/ml (**b**). CFU, Colony-Forming Units

After confirming that CQ exhibited antifungal activity, it was deemed of interest to investigate if CQ simply inhibited fungal growth (is fungistatic) or killed fungi (is fungicidal). The minimum fungicidal concentration (MFC) of CQ was 3 μg/ml against *C. albicans*, which demonstrated that CQ had fungicidal activity (MFC/MIC≤3) for *C. albicans*. It was also confirmed through the time-kill curve again (Fig. 1). The starting inoculum concentration was 5 × 10^5^ CFU/ml (Fig. 1a) and 5 × 10^6^ CFU/ml (Fig. 1b) respectively. Eight μg/ml (8 × MIC) of CQ did generate an equal or more than 3log_10_ reduction in fungal CFU after 24- or 36- or 48-h’ treatment compared to untreated cells, which demonstrated the fungicidal of CQ. Similarly, it was also observed in 4 μg/ml (4 × MIC) CQ group after 48 h’ treatment. These results indicated that CQ was a fungicidal agent (particularly against *C. albicans*).

### Clioquinol interfered with yeast-hyphae transition in *C. albicans*

**Fig. 2:**
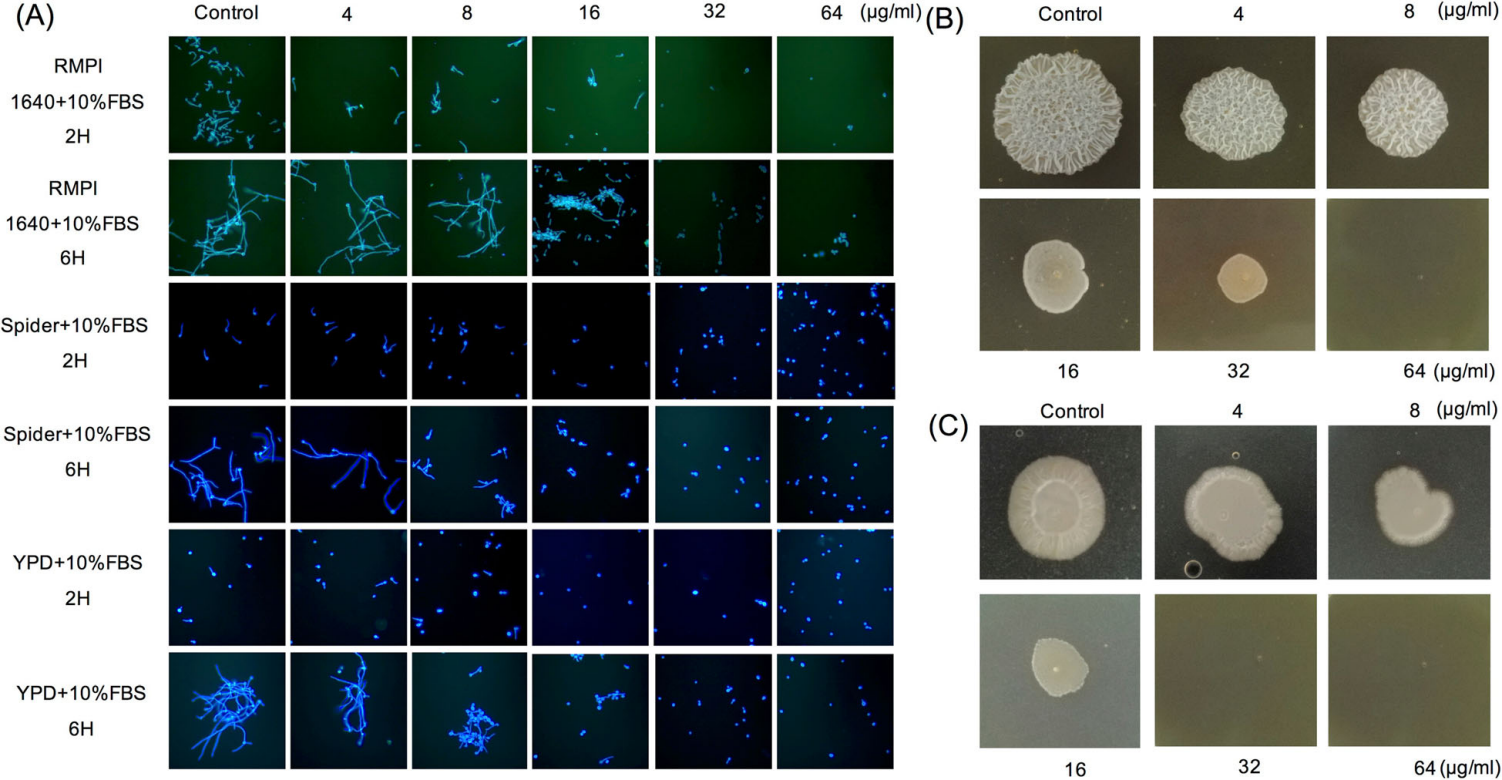
The effect of clioquinol in hyphae formation. **a** The morphology of fungal cells under microscopy treated with clioquinol in different liquid media; **b** Colony morphology of fungal cells treated with clioquinol in YPD solid media (containing 10%FBS); **c** Colony morphology of fungal cells treated with clioquinol in Spider solid media (containing 10%FBS). YPD, yeast extract peptone dextrose; FBS, fetal bovine serum

We examined the effect of CQ on hyphae growth in three different liquid media at 37 °C (Fig. 2a), the formation of hyphae was inhibited in a concentration-dependent manner. After 2 h’ treatment, hyphae formation was significantly reduced when cells were treated with 8 or 16 μg/ml of CQ in RMPI 1640 and Spider medium (containing 10% fetal bovine serum (FBS)). Meantime almost no hyphae were formed at concentrations of 32 and 64 μg/ml. The trend was more obvious in yeast peptone dextrose (YPD) medium (containing 10% FBS) with almost no formation of hyphae in lower concentrations-16 μg/ml. When prolonging incubation time to 6 h, untreated cultures exhibited branched filamentous cells characteristic of true hyphae. However, cells treated with 32 and 64 μg/ml of CQ exhibited more than 95% inhibition of hyphae formation in three different media. It was also observed in lower concentration-16 μg/ml in YPD and Spider medium (containing 10% FBS).

The similar results were observed in a solid growth media too (Fig. 2b&c). It was found that 16 μg/ml CQ was sufficient to abrogate filamentation in YPD and Spider medium (containing 10% FBS). According to the results of *C. albicans* in liquid and solid medium, we found that CQ could inhibit yeast-hyphae transition in a concentration dependent manner.

### Clioquinol inhibited biofilm formation

**Fig. 3:**
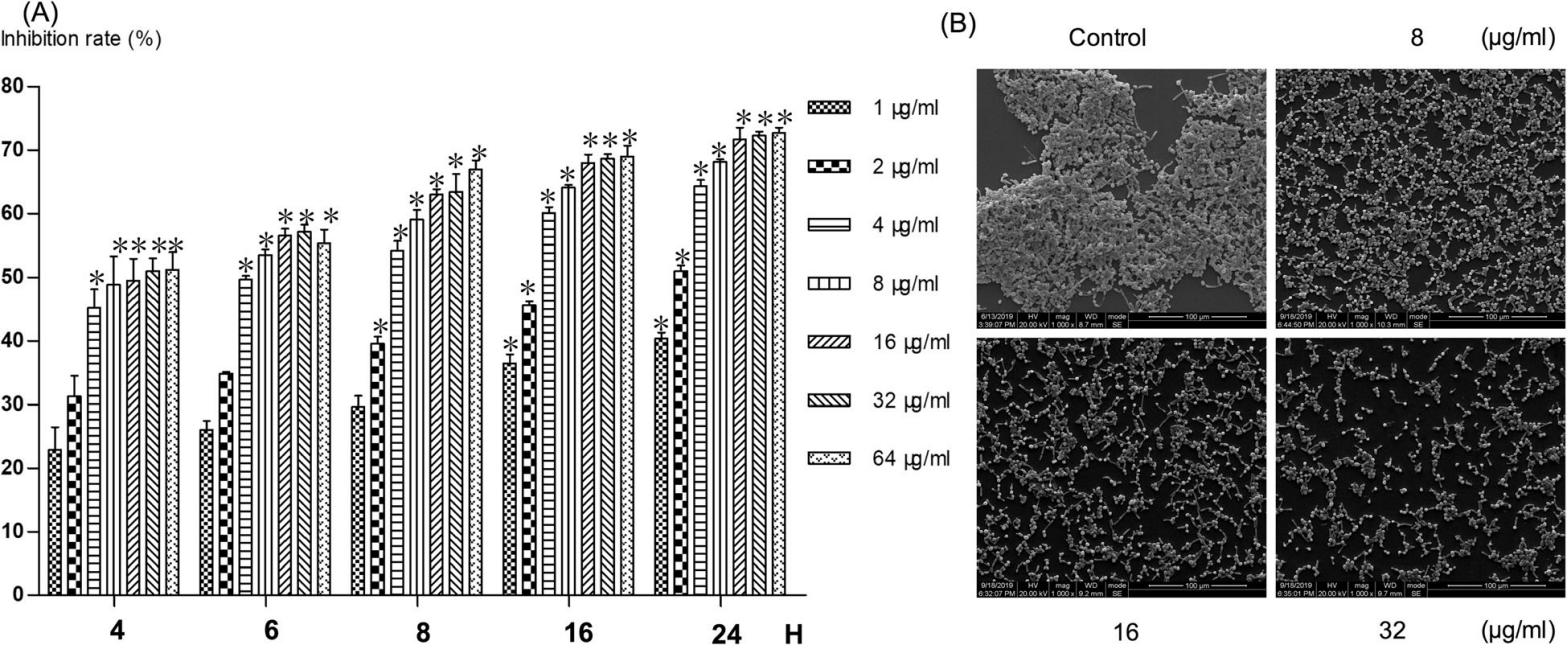
The effect of clioquinol in biofilm formation using XTT assay. **a** The inhibition rate increased with the increasing concentration or incubation time; **b** Biofilm formation was inhibited under scanning electron microscope. XTT, 2,3-bis (2-methoxy-4-nitro-5-sulfo-phenyl)-2H-tetrazolium-5-carboxanilide; *, *p* < 0.05

In this study, we investigated the effect of CQ on fungal biofilms formation through 2,3-bis (2-methoxy-4-nitro-5-sulfo-phenyl)-2H-tetrazolium-5-carboxanilide (XTT) reduction assay and scanning electron microscope (SEM) observation (Fig. 3). The effect was concentration-dependent, as reflected by a progressive increase in inhibition rate, with increasing concentrations of CQ. The inhibition rate increased from 22.9% at 1 μg/ml to 51.7% at 64 μg/ml after 4 h’s treatment. The similar trend was also observed after 6 h’s, 8 h’s, 16 h’s and 24 h’s treatment. The effect was also time-dependent. The inhibition rate increased from 22.9% after 4 h’s treatment to 40.4% after 24 h’s treatment when cells were treated with 1 μg/ml CQ. The similar trend was also observed in other concentrations. After 24 h’s treatment, the inhibition rate was more than 70% at 16, 32 and 64 μg/ml of CQ. And most differences between CQ treated cells and untreated cells were statistically significant (Fig. 3a).

The anti-biofilm effect of CQ was further confirmed through SEM. The formation of biofilms was inhibited by CQ in a concentration-dependent manner. After 8 h’s treatment, the biofilm formation was significantly inhibited at different concentrations of CQ (Fig. 3b).

### Clioquinol did not damage cell wall directly

Damage to the components of cell wall from antifungal agents influences the normal growth of fungal cells. However, cells will continue to grow in the presence of a suitable stabilizer (such as sorbitol) in the medium, it will lead to an increase of MIC values. It was observed in caspofungin (positive control). When *C. albicans* was treated with CQ in a medium supplemented with sorbitol, MICs did not increase after 2 or 7 days of incubation compared to MIC in medium without sorbitol (Table 1). The results demonstrated that CQ could not damage the cell wall.

**Table 1:** Sorbitol protection assay of *C. albicans*

MIC (μg/ml)	Clioquinol				Caspofungin			
	2 d		7 d		2 d		7 d	
	Sorbitol (−)	Sorbitol (0.8 M)	Sorbitol (−)	Sorbitol (0.8 M)	Sorbitol (−)	Sorbitol (0.8 M)	Sorbitol (−)	Sorbitol (0.8 M)
SC5314	1	1	1	1	0.5	0.5	0.5	4
ATCC10231	2	2	2	2	0.25	0.5	0.25	4
Clinical strain 1	1	1	1	1	0.25	1	0.25	4
Clinical strain 2	1	1	1	1	0.5	1	0.5	8

Note: *MIC* minimum inhibitory concentration

### Clioquinol induced cell membrane disruption and depolarization

**Fig. 4:**
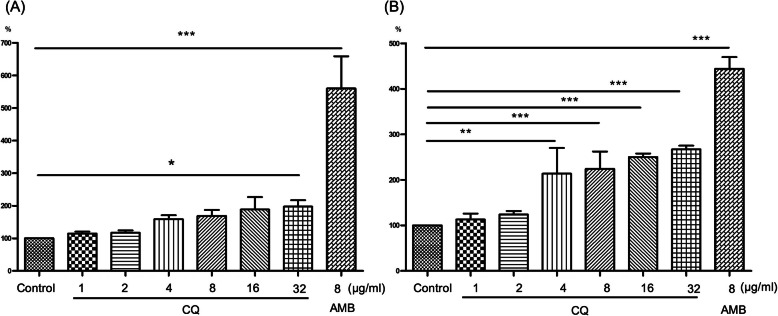
The effect of clioquinol in cell membrane. **a** Using propidium iodide influx assay to evaluate the influence on plasma membrane integrity; **b** Using DiBAC_4_(3) to evaluate the membrane potential. *, *p* < 0.05; **, *p* < 0.001; ***, *p* < 0.0001; DiBAC_4_(3), bis-(1,3-dibutylbarbituric acid) trimethine oxonol; CQ, clioquinol; AMB, amphotericin B

We used propidium iodide (PI) influx assay to investigate the effect of CQ on cell membrane integrity. Because of its large molecular weight, propidium iodide can only enter through compromised membranes. After 12 h of incubation with CQ (1, 2, 4, 8, 16 and 32 μg/ml), the uptake of PI increased by 14.6, 17.0, 58.8, 68.5, 82.6 and 96.9%, respectively (Fig. 4a). But only the increase of 32 μg/ml CQ treated cells was statistically significant (*p* < 0.05). In contrast, amphotericin B (8 μg/mL) showed a significant fluorescence increase (*p* < 0.0001) in greater than 400%. The difference between CQ (32 μg/mL) and amphotericin B (8 μg/mL) was also statistically significant (*p* < 0.0001). Accordingly, we deduced that CQ may result in fungal membrane disruption at relatively high concentration (32 μg/mL).

To further confirm whether CQ affected the normal functions of the fungal plasma membrane, especially in relatively low concentration, cell membrane potential was investigated using bis-(1,3-dibutylbarbituric acid) trimethine oxonol (DiBAC_4_(3)). The cell membrane potential is consistently maintained in normal conditions, and it is required for cell survival and the electrogenic transport of nutrients. However, cells with cell membrane damage will gradually lose the normal membrane potential and become depolarized. The entry of the membrane potential indicator, DiBAC4(3), into depolarized cells leads to an increase in fluorescence intensity. We observed the concentration-dependent effect of CQ in membrane depolarization. CQ increased DiBAC_4_(3) fluorescence intensity by 12.8, 23.8, 113.7, 124.2, 147.8 and 167.2% at different concentrations (1, 2, 4, 8, 16 and 32 μg/ml) compared with untreated cells, respectively (Fig. 4b). And the differences between cells treated with CQ (4, 8, 16 and 32 μg/ml) and untreated cells were statistically significant (*p* < 0.01 or 0.0001). Amphotericin B application resulted in an increase of 344.1% (*p* < 0.0001). These results supported the influence of low concentration of CQ on the normal function of the fungal cell membrane.

If the activity of antifungal agents is caused by binding to ergosterol, the exogenous ergosterol will prevent the binding to the cell membrane’s ergosterol, leading to an increase of MIC values. In fact, the MIC values of amphotericin B (positive control) against *C. albicans* increased from 0.5 or 1 to 32 μg /ml dependent on different strains. However, MICs of CQ against *C. albicans* did not increase after 2 or 7 days of incubation compared to the MIC in medium without ergosterol (Table 2). The results demonstrated that CQ could not inhibit the growth of *C. albicans* by binding to ergosterol as amphotericin B.

**Table 2:** Ergosterol binding assay of *C. albicans*

MIC (μg/ml)	Clioquinol				Amphotericin B			
	2 d		7 d		2 d		7 d	
	Ergosterol (−)	Ergosterol (+)	Ergosterol (−)	Ergosterol (+)	Ergosterol (−)	Ergosterol (+)	Ergosterol (−)	Ergosterol (+)
SC5314	1	1	1	1	1	32	1	32
ATCC10231	2	2	2	2	0.5	32	0.5	32
Clinical strain 1	1	1	1	1	0.5	32	0.5	32
Clinical strain 2	1	1	1	1	1	32	1	32

Note: *MIC* minimum inhibitory concentration

### Clioquinol disrupted metal ion homeostasis

**Fig. 5:**
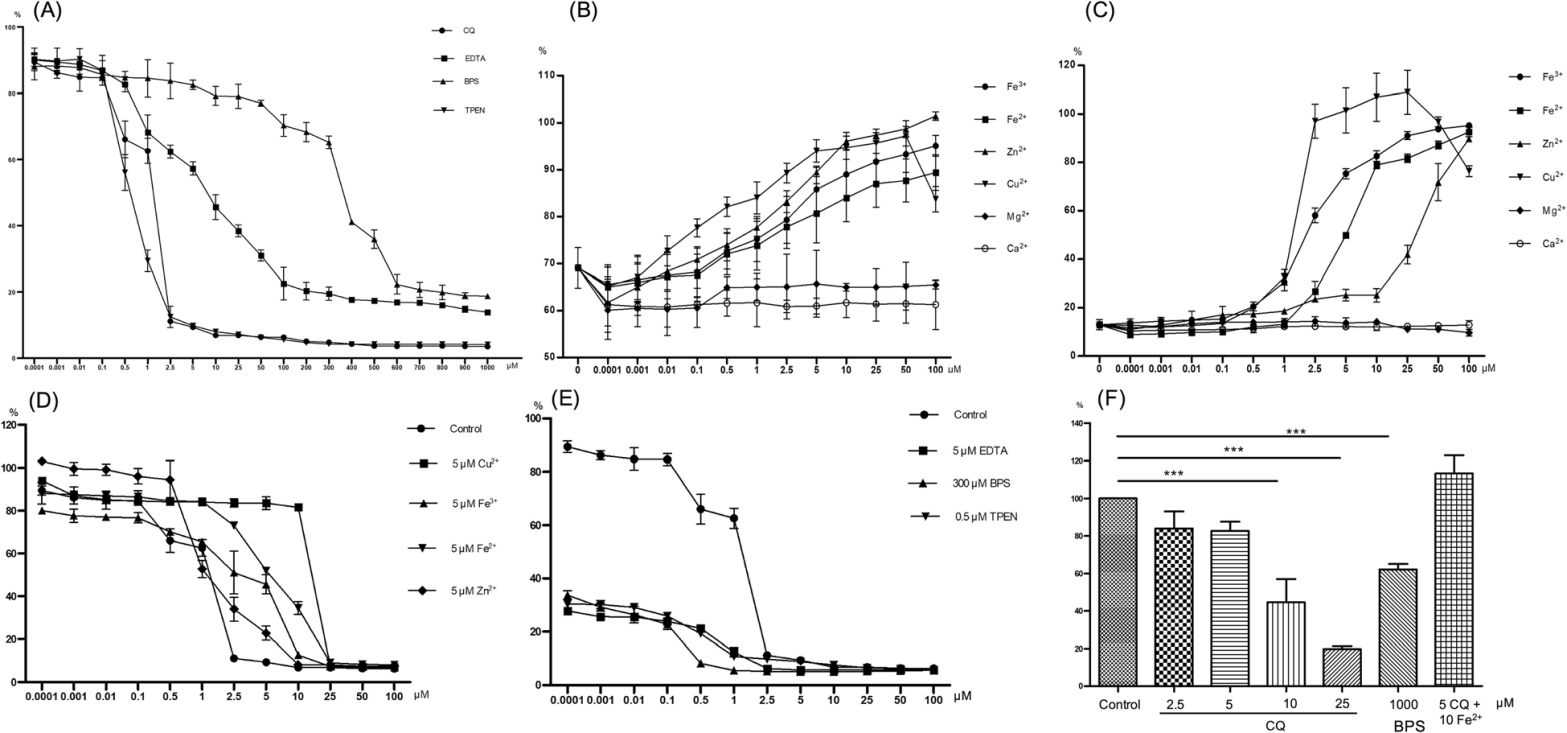
The influence of clioquinol on ion homeostasis. The optical density (OD) at 600 nm of fungal cells in RMPI 1640 medium without addition of clioquinol, exogenous metal ions, exogenous metal chelators was used as standard value in (**a** to **e**). The OD values of experiment groups were compared to standard value to obtain the relative value. **a** The antifungal activity of clioquinol and metal chelators; **b** The influence of exogenous metal ions in antifungal activity of 1μM clioquinol; **c** The influence of exogenous metal ions in antifungal activity of 5 μM clioquinol; **d** The influence of exogenous metal ions in MIC value of clioquinol; **e** The influence of exogenous metal chelators in MIC value of clioquinol; **f** Cellular labile ferrous iron level in clioquinol-treated fungal cells. Fluorescence intensity of fungal cells in YPD medium without addition of clioquinol, exogenous metal ions, exogenous metal chelators was used as standard value and the fluorescence intensities of experiment groups was compared to standard value to obtain the relative value. MIC, the minimum inhibitory concentration; EDTA, ethylenediamine tetraacetic acid; TPEN, N,N,N′,N′-Tetrakis-(2-pyridylmethyl) ethylenediamine; BPS, basophenanthrolinedisulfonate disodium salt; YPD: Yeast peptone dextrose; ***, *p* < 0.0001

The MIC_80_ values of CQ, ethylenediamine tetraacetic acid (EDTA) (chelator for multiple metals), N,N,N′,N′-Tetrakis-(2-pyridylmethyl) ethylenediamine (TPEN) (chelator for zinc) and basophenanthrolinedisulfonate disodium salt (BPS) (chelator for iron) was 2.5, 200, 2.5 and 700 μM, respectively (Fig. 5a). While the inhibitory effect of heavy metal ions was very weak (inhibition rate was lower than 8%) when the concentrations were equal or lower than 100 μM (except 50 μM or higher concentration of copper ion).

CQ can work as metal chelator or ionophore dependent on different conditions. If CQ inhibits the growth of *C. albicans* as a metal ionophore, the antifungal effects of 1 μM CQ (inhibition rate was about 40%) will increase by exogenous addition of metal ions (Fe^3+^ or Fe^2+^ or Cu^2+^ or Zn^2+^ or Mg^2+^ or Ca^2+^) because more metal ions are transported into cells and lead to excessive metal ions accumulation in cells, which leads to inhibition of fungal cells growth. However, the inhibition rate did not increase with the exogenous addition of metal ions in the presence of 1 μM CQ (Fig. 5b).

On the contrary, if CQ inhibits the growth of *C. albicans* as a metal chelator, the antifungal effects of 5 μM CQ (inhibition rate was about 80%) will be reversed by exogenous addition of metal ions (Fe^3+^ or Fe^2+^ or Cu^2+^ or Zn^2+^ or Mg^2+^ or Ca^2+^) because chelated metal ions will be supplemented with new metal ions. Indeed, the antifungal effects of CQ were reversed by exogenous addition of zinc or copper or ferrous iron or ferric iron to the growth medium in the presence of 5 μM CQ (Fig. 5c). Copper ions were most effective in reversing the growth-inhibitory effects of the CQ, with a fungal growth rate of more than 90% in the presence of 2.5 μM Cu^2+^ compared to control cells. Addition of ferric irons had a modest effect on the antifungal activity of CQ, with a fungal growth rate of more than 50% in the presence of 2.5 μM Fe^3+^ compared to control cells. As for Zn^2+^ and Fe^2+^, the fungal growth rate was approximately 20% at 2.5 μM. When the concentration increased to 10 μM, the growth rate was increased to about 80% for Fe^3+^ and Fe^2+^. However, the concentration of Zn^2+^ required to obtain 80% growth rate was more than 50 μM. The addition of magnesium or calcium did not affect the antifungal activity of CQ. These results supported that CQ could inhibit the growth of *C. albicans* as metal chelator for zinc, copper and iron.

We also found that the MIC value of CQ increased when exogenous metal ions (5 μM Fe^3+^ or Fe^2+^ or Cu^2+^ or Zn^2+^) were added to the medium because CQ needed to chelate more metal ions to inhibit the growth of fungal cells (Fig. 5d). The value increased from 2.5 μM to 25 μM in medium added with Fe^2+^ or Cu^2+^, to 10 μM in medium added with Fe^3+^ or Zn^2+^. On the contrary, the MIC value of CQ decreased when exogenous metal chelators (5 μM EDTA or 300 μM BPS or 0.5 μM TPEN) were added to the medium because less metal ions were needed to be chelated by CQ to inhibit the growth of fungal cells (Fig. 5e). The value decreased from 2.5 μM to 1 μM for medium added with EDTA, to 0.5 μM for medium added with BPS or TPEN. These two experiments also confirmed that the mechanism of CQ was related to chelating metal ions.

Then we used FeRhoNox™-1 to measure cellular labile ferrous iron level. FeRhoNox™-1 fluorescence upon the reaction of Fe^2+^ ions but does not react with other ions. We found cellular ferrous iron level decreased in a concentration-dependent manner. Fluorescence intensity decreased to 84, 82.6, 44.7 and 19.8% after treatment at different concentrations (2.5, 5, 10 or 25 μM) of CQ compared to untreated cells, respectively (Fig. 5f). This trend was also observed in BPS (positive control, iron chelator). The differences between the fluorescence intensity of cells treated with CQ at different concentrations (10 and 25 μM) or 1000 μM BPS and that of untreated cells were statistically significant (*p* < 0.0001). We also observed exogenous addition of ferrous iron (10 μM Fe^2+^) to the medium could reverse the decrease of ferrous iron level in the presence of 5 μM CQ. It confirmed that CQ could decrease the cellular ferrous iron level, which also supported the metal chelating properties of CQ.

## Discussion

Although most *Candida spp.* infections were not lethal, the mortality rate of candidemia is high. However, only several classes of antifungal agents are available in clinical work. And lots of fungi share similar metabolic pathways and necessary cellular processes with human beings, leading to the lack of selective fungal targets [1]. Hence, as per the above reason and the increasing drug-resistant strains, there is urgent to develop new antifungal agents with novel drug targets.

CQ was marketed in 1934 by Ciba-Geigy (Now Novartis) as an antimicrobial agent. Although the anti-neurodegenerative diseases and anti-malignancy mechanisms of CQ are widely studied in previous literature [2, 4–7], mechanism of CQ in inhibition of fungal cells remains poorly understood [12–14].

In present study, we found that CQ exhibited fungicidal activity for *C. albicans* in time and concentration dependent manner. For this strain, 4 and 8 μg/ml CQ showed a fungicidal effect when evaluated at 48 h, and the 8 μg/ml CQ had the same effect in less time-24 h. Similar results were also observed in Yan et al’s study [12]. However, the fungicidal activity for the *C. albicans* isolate was not observed in Pippi et al’s study [9]. The strains used in three studies were different, which may partly explain the inconsistent results. Hence, more strains and experiments are needed to confirm the fungicidal activity of CQ.

Pippi et al. [13] reported that CQ inhibited the formation of pseudohyphae in *C. albicans* while pseudohyphae was just intermediate form between yeast and true hyphae, and they did not observe the fungal morphology changes under hyphae-inducing conditions. In present study, we found that true hyphae formation could also be inhibited by CQ in concentration-dependent manner in both solid and liquid media. Sixteen μg/ml of CQ was sufficient to completely inhibit filamentation under most hypha-inducing conditions. Actually 8 μg/ml of CQ could inhibit the hyphae formation in some media, such as Spider or YPD liquid media containing 10% FBS.

Pippi et al. [14] observed the effect on biofilm formation after 48 h’ incubation, which was too long for observe the effect on biofilm formation. In fact, action time of CQ was much shorter than 48 h in clinical work and exponential growth phase of *C. albicans* was much shorter than 48 h (about 16 to 18 h). Early biofilm was formed as early as 1 h’s incubation period. Meanwhile, evaluation in only one point of time made the results unconvincing. Based on these, incubation periods of biofilm formation in most biofilm study were less than 24 h and evaluation was conducted in different incubation intervals to make the results more convincing [17, 18].

Hence, we conducted our investigation of CQ on *C. albicans* biofilms formation using both qualitative and quantitative methods in different incubation periods. Several methods are available to quantitatively assess the viability of *C. albicans* biofilms. However, Taff HT et al. [22] clearly demonstrated that XTT reduction assay provided the most reproducible and accurate measurement. The inhibition on biofilm formation was in concentration and time dependent manner, as reflected by a progressive increase in inhibition rate, with increasing concentrations of CQ or time of treatment. SEM was employed as a qualitative analytical tool to reveal the morphology and architecture of *Candida* biofilms, which also demonstrated the inhibitory effect of CQ on biofilm formation in concentration dependent manner again. These results above demonstrated that CQ inhibit the true hyphae formation in multiple hyphae-inducing conditions and biofilm formation in concentration and time dependent manner.

Current antifungal drug inhibits fungal cell membrane/wall synthesis or directly bind to the cell membrane. To investigate the effect on cell wall, sorbitol protection assay was used [23]. Unchanged MIC value of CQ after adding sorbitol demonstrated CQ could not damage cell wall directly. This result was opposite to the results of Pippi et al. [13]. More studies maybe required to explain the influence of CQ on fungal cell wall except for sorbitol protection assay.

As for the effect on cell membrane, Yan et al. [12] and Pippi et al. [13] reported that CQ could not disrupt membrane in *S. cerevisiae* and *C. albicans*, respectively. In the present study, we found that high concentration (32 μg/ml) of CQ could disrupt the cell membrane but no obvious disruption was seen in lower concentration. Actually, the concentrations of CQ used in Yan and Pippi’s study were the concentrations of MIC values while CQ in MIC value of concentration (1 μg/ml) in our study did not disrupt membrane either. Based on these findings, we presumed that CQ could disrupt cell membrane in high concentration.

However, effect of relatively low concentration of CQ on cell membrane is still not well understood. In present study, we found that 4 μg/ml or higher concentrations of CQ could cause depolarization of the membrane and the effect was concentration dependent. Normal membrane potential is necessary for cell survival. Membrane depolarization could be observed before the disruption of cell membrane integrity. The depolarization is related to cell viability decrease and impairment of essential cell processes. Finally, cell death can be observed [24, 25]. Hence, low concentration of CQ (4 μg/ml) could influence the normal function of membrane through membrane depolarization and then lead to fungal inhibition. These two assay demonstrated that low concentration of CQ could affect the normal function of membrane while high concentration of CQ could disrupt the membrane integrity.

If the activity of the antifungal agent is a consequence of binding to ergosterol, external ergosterol would prevent the binding, consequently, the MIC of the antifungal agent would increase [26]. However, unchanged MICs for CQ in the presence of exogenous ergosterol suggested that they do not act by binding to the membrane ergosterol, which was consistent to the results of Pippi et al. [13].

CQ can form relatively stable complexes with zinc, iron and copper ions as metal chelators [27, 28], which means that it may exhibit the potential to influence ion homeostasis. However, the metal chelating properties of CQ are still debated because some researchers found CQ could act as metal ionophore rather than metal chelators, especially for zinc [4].

In the present study, we found several other metal chelators (EDTA, TPEN and BPS) exhibited antifungal activity, which demonstrated that the influence in ion homeostasis could lead to inhibition of *C. albicans* growth. The antifungal effect of 5 μM CQ (inhibition rate was about 80%) could be reversed by exogenous addition of iron, zinc and copper. Copper ions were most effective in reversing the growth-inhibitory effects. Addition of ferric irons had a modest effect while ferrous iron and zinc had a relatively weaker effect. We also found noticeable increase in MIC value was observed for CQ in the presence of exogenous metal ions while noticeable decrease in MIC value was observed in the presence of exogenous metal chelators. The cellular labile ferrous iron level of CQ-treated cells decreased in concentration-dependent manner, which was also observed in iron chelator-BPS treated cells. These approaches demonstrated that CQ exerted its antifungal effect in *C. albicans* by targeting metal ion homeostasis as metal chelator for zinc, iron and copper.

## Conclusions

In summary, CQ exhibited fungistatic and fungicidal activity against *C. albicans.* CQ inhibited true hyphae formation in concentration-dependent manner in multiple hyphae-inducing conditions. The concentration and time dependent anti-biofilm activity of CQ was confirmed quantitatively and qualitatively in different incubation periods. Further investigations of CQ’s antifungal mechanism demonstrated that CQ disrupted cell membrane directly in high concentration and induced depolarization of the membrane in low concentration. However, CQ did not bind to ergosterol to influence the cell membrane. Unlike the current antifungal agents, CQ also interfered with ion homeostasis in *C. albicans* to inhibit growth of fungi, which was quite different from current antifungal agents. Although more experiments are needed, clioquinol could be a new promising antifungal agent with novel target in the future based on results of this study.

## Methods

### Strain, cultivation and chemicals

*C. albicans* SC5314, ATCC10231 and 2 clinical strains were routinely cultured in Sabouraud dextrose agar (SDA) at 30 °C. To prepare a cell suspension, a single colony was inoculated into yeast peptone dextrose (YPD, Aoboxing, China) liquid medium and incubated for 16 to 18 h at 30 °C with agitation (150 rpm). Clioquinol (CQ) was purchased from Tokyo Kasei Kogyo® (Tokyo, Japan) as standard powder. Amphotericin B (AMB), terbinafine (TBF), itraconazole (ITR), fluconazole (FLC), ketoconazole (KTZ) and caspofungin (CSF) were purchased from Sigma Aldrich® (St. Louis, MO, USA). All agents were diluted in dimethyl sulfoxide (DMSO) at concentration of 25,600 μg/ml as stock solutions and stored at − 80 °C.

### Antifungal activity of clioquinol

#### The broth microdilution assay

The minimum inhibitory concentration (MIC) of CQ and other common antifungal agents were determined according to the guidelines provided by the Clinical and Laboratory Standards Institute for yeasts (M27-A3) [10]. Plates were incubated at 30 °C for 48 h. MIC_100_ and MIC_50_ were used to evaluate the antifungal activity of AMB and other antifungal agents, respectively.

#### The minimum fungicidal concentration (MFC)

The MFC of CQ against *C. albicans* SC5314 was determined by two methods: (1) After measuring MIC, 5 μl suspension form wells without visible fungal growth was inoculated in new fresh RMPI 1640 (Gibco, USA) medium (200 μl) for 24 h at 30 °C. (2) After measuring MIC, 100 μl suspension form wells without visible fungal growth was inoculated in SDA plate for 48 h at 30 °C. MFC was defined as the minimal concentration of CQ required to kill 99.9% organism.

#### Time-kill assay

Concentration of *C. albicans* SC5314 cell suspension was adjusted to 5 × 10^5^ or 5 × 10^6^ cells/ml by Microplate Reader (Eon, Bioterk, USA) and confirmed by haemocytometric counting using fresh RMPI-1640 medium. The suspension was exposed to different concentrations (1 to 8 μg/ml) of CQ or DMSO (control group) in RMPI 1640 medium. A sample (100 μl) was obtained at 0, 12, 24, 36 and 48 h after incubation at 30 °C with agitation (150 rpm) and subsequently serially diluted in phosphate buffer saline (PBS). An aliquot of each dilution was transferred to SDA agar plates and incubated at 30 °C for 48 h. The number of Colony-Forming Units (CFU) was subsequently enumerated.

### Effect of clioquinol on *C. albicans* yeast-hyphae transition

#### Solid media

Cell suspension of *C. albicans* SC5314 (1 × 10^7^ cells/ml) was inoculated in the center of Spider or yeast peptone dextrose (YPD) plates containing 10% fetal bovine serum (FBS, Gibco, USA) supplemented with DMSO or different concentrations (4 to 64 μg/ml) of CQ. The plates were incubated at 37 °C for 4 days and the morphology of fungal colony was photographed using a digital camera.

#### Liquid media

Cell suspension of *C. albicans* SC5314 (1 × 10^6^ cells/ml) was inoculated in Spider or YPD or RMPI 1640 liquid medium containing 10% FBS supplemented with DMSO or different concentrations (4 to 64 μg/ml) of CQ. Cells were grown at 37 °C with agitation (250 rpm) for 6 h. Morphology of fungal cells was observed under optical microscope (Olympus CX43, Guangzhou, China) after 2- and 6-h’ treatment.

### Effect of clioquinol on *C. albicans* biofilm formation

Concentration of *C. albicans* SC5314 cell suspension was adjusted to 1 × 10^7^ cells/ml using fresh RMPI-1640 medium containing 10% FBS. Cell suspension of *C. albicans* (100 μl) was transferred into 96-well plates that were pretreated with RMPI 1640 containing 10% FBS for 24 h at 37 °C and incubated for 1.5 h at 37 °C with agitation (75 rpm). After the adhesion phase, the liquid was aspirated and each well was washed with PBS to remove loosely attached cells. Fresh RMPI 1640 medium with 10% FBS (200 μl) containing different concentrations (1 to 64 μg/ml) of CQ were added to each well and the plate was further incubated at 37 °C for 24 h with agitation (75 rpm). Blank control group was set at the same time. Measure biofilm activity after 4, 6, 8, 16 and 24 h’ treatment.

### Quantitative analysis-XTT reduction assay [17, 19]

The supernatant was aspirated and the wells were washed twice with PBS at each time of measurement. The fungal cell viability was determined using colorimetric 2,3-bis (2-methoxy-4-nitro-5-sulfo-phenyl)-2H-tetrazolium-5-carboxanilide (XTT) reduction assay that measures the activity of mitochondrial dehydrogenase. XTT (Macklin, China) solution (1 mg/ml) was prepared by dissolving XTT powder in PBS, and the solution was filter-sterilized (0.22 mm pore size filter). XTT solution (40 μl) was mixed with freshly prepared menadione (Meilun, China) solution (0.4 mM; 2 μl) at 20:1 (v/v) immediately prior to the assay. Thereafter, PBS (158 μl) was mixed with XTT-menadione solution (42 μl) and transferred to each well containing pre-washed biofilms, and incubated in the dark for 2 h at 37 °C. After the incubation, the colored supernatant (100 μl) was transferred to new microtiter plates, and the optical density (OD) of the supernatant was measured at 492 nm with a microplate reader (Eon, Bioterk, USA). All assays were carried out in triplicate on three different occasions. One way ANOVA and Dunnett-t test were used to compare the differences between different groups using IBM SPSS Statistics software (version 22, 2013; IBM Corporation, New York, USA).

### Qualitative analysis-scanning electron microscopy [17]

Flat-bottomed 12-well polystyrene microtiter plates were used to prepare biofilms as described above. Presterilized coverslips were placed in the wells of the plates, *C. albicans* SC5314 biofilm was prepared as described above. After 8-h treatment, the coverslips were washed with PBS and placed in 2.5% glutaraldehyde for 4 h at 4 °C. Samples were subsequently dehydrated in a series of ethanol solutions (50% for 5 min, 70% for 5 min, 90% for 5 min and 100% for 5 min), and sputtered coating with gold. The surface topographies of the *C. albicans* biofilms were viewed with a scanning electron microscope (FEI Insepct F, Hillsboro, USA).

### Effect of clioquinol on *C. albicans* cell wall-sorbitol protection assay [23]

The MICs of CQ and CSF (positive control) against *C. albicans* SC5314 were determined in the presence and absence of sorbitol (0.8 M) by the microdilution broth method. Probes were incubated at 28 °C for 7 days and evaluation was conducted after 2- and 7-days’ incubation.

### Effect of clioquinol on *C. albicans* cell membrane

#### Influence on cell membrane integrity- propidium iodide influx assay

Concentration of *C. albicans* cell suspension was adjusted to 2 × 10^7^ cells/ml using fresh YPD medium. The cells were treated with various concentrations of CQ (0 to 32 μg/mL) and AMB (8 μg/mL) at 30 °C for 12 h with agitation (150 rpm). Then the cells were harvested, resuspended in PBS and stained with 20 μg/mL propidium iodide (PI, Solarbio, China) for 60 min at 30 °C with agitation (150 rpm). The cells were analyzed using Fluorescence microplate reader (Synergy Mx, Biotex, USA) with excitation wavelength of 535 nm and an emission wavelength of 615 nm to evaluate the damage to the plasma membrane. One way ANOVA and Dunnett-t test were used to compare the differences between different groups using IBM SPSS Statistics software (version 22, 2013; IBM Corporation, New York, USA).

#### Influence on cell membrane potential-membrane depolarization assay

The cells were treated as above. Subsequently, the cells were treated with 5 μg/mL bis-(1,3-dibutylbarbituric acid) trimethine oxonol [DiBAC_4_(3), AAT Bioquest, USA] for 60 min at 30 °C with agitation (150 rpm) and analyzed using Fluorescence microplate reader (Synergy Mx, Biotex, USA) with excitation wavelength of 490 nm and an emission wavelength of 525 nm. One way ANOVA and Dunnett-t test were used to compare the differences between different groups using IBM SPSS Statistics software (version 22, 2013; IBM Corporation, New York, USA).

#### Ergosterol assay [26]

Forty mg ergosterol was dissolved in 0.5 ml DMSO, then Tween-80 was added to make the emulsion. Next the emulsion was dissolved in 100 ml RMPI 1640 medium to produce the working solution (400 μg/mL). The MICs of CQ and AMB (positive control) against *C. albicans* SC5314 were determined by the microdilution broth method in the presence and absence of exogenous ergosterol. Probes were incubated at 28 °C for 7 days and evaluation was conducted after 2- and 7-days’ incubation.

### Effect of clioquinol on ion homeostasis

#### Antifungal activity of clioquinol, metal ions and metal chelators

Antifungal activity of CQ, metal chelators [ethylenediamine tetraacetic acid (EDTA), N,N,N′,N′-Tetrakis-(2-pyridylmethyl) ethylenediamine (TPEN, MedChemExpress, USA), basophenanthrolinedisulfonate disodium salt (BPS, Sigma Aldrich, USA)], heavy metals (ZnSO_4_, CuSO_4_, FeCl_3_, (NH_4_)_2_Fe(SO4)_2_, MgCl_2_ and CaCl_2_) was measured using microdilution broth assay with a few modifications. Cell suspension was diluted to the final concentration of 5 × 10^5^ cells/mL. Cell suspension (100 μL) plus substances above (100 μL, 0.0001 to 100 μM) was added to microtiter plates for 24 h. Fungal growth was determined using microtitre plate reader (Eon, Bioterk, USA) by optical density at 600 nm. MIC_80_ was defined as the lowest compound concentration that resulted in at least 80% growth inhibition.

#### Influence of exogenous metal ions to antifungal activity of clioquinol

The growth effect of exogenous addition of various metal ions was evaluated by performing the broth microdilution assay described above in the presence of 1 or 5 μM CQ with increasing concentration (0.0001 to 100 μM) of ZnSO_4_, CuSO_4_, FeCl_3_, (NH_4_)_2_Fe(SO4)_2_, MgCl_2_ or CaCl_2_.

#### Influence of exogenous metal ions or metal chelators to MIC of clioquinol

The MIC of clioquinol was measured using the broth microdilution assay described above in the presence of 5 μM metal ions (ZnSO_4_, CuSO_4_, FeCl_3_, (NH_4_)_2_Fe(SO4)_2_) or 5 μM EDTA or 300 μM BPS or 0.5 μM TPEN.

#### Cellular labile ferrous iron level

Concentration of *C. albicans* SC5314 cell suspension was adjusted 2 × 10^7^ cells/ml using fresh YPD. The cells were treated with various concentrations of clioquinol (0 to 25 μM) or clioquinol (5 μM) plus (NH_4_)_2_Fe(SO4)_2_) (10 μM) or BPS (1000 μM) at 30 °C for 16 h with agitation (150 rpm). After incubation, the cells were harvested, resuspended in PBS and stained with 2.5 μM FeRhoNox™-1 (Goryo, Sapporo, Japan) for 60 min at 37 °C with agitation (150 rpm). The cells were analyzed using Fluorescence microplate reader (Synergy Mx, Biotex, USA) with excitation wavelength of 535 nm and an emission wavelength of 570 nm to evaluate the cellular labile ferrous iron level. One way ANOVA and Dunnett-t test were used to compare the differences between different groups using IBM SPSS Statistics software (version 22, 2013; IBM Corporation, New York, USA).

## Data Availability

The datasets used and analyzed during the current study available from the corresponding author on reasonable request.

## Abbreviations

AMB: Amphotericin B
BPS: Basophenanthrolinedisulfonate disodium salt
CQ: Clioquinol
CSF: caspofungin
DMSO: Dimethyl sulfoxide
DiBAC_4_(3): Bis-(1,3-dibutylbarbituric acid) trimethine oxonol
EDTA: Ethylenediamine tetraacetic acid
FBS: Fetal bovine serum
FLC: Fluconazole
ITR: Itraconazole
KTZ: Ketoconazole
MFC: Minimum fungicidal concentration
MIC: Minimum inhibitory concentration
PBS: phosphate buffer saline
PI: Propidium iodide
SDA: Sabouraud dextrose agar
SEM: Scanning electron microscope
TPEN: N,N,N′,N′-Tetrakis-(2-pyridylmethyl) ethylenediamine
TBF: Terbinafine
XTT: 2,3-bis (2-methoxy-4-nitro-5-sulfo-phenyl)-2H-tetrazolium-5-carboxanilide
YPD: Yeast peptone dextrose
